# Metabolomic profiling of macrophages determines the discrete metabolomic signature and metabolomic interactome triggered by polarising immune stimuli

**DOI:** 10.1371/journal.pone.0194126

**Published:** 2018-03-14

**Authors:** Kevin M. Rattigan, Andrew W. Pountain, Clement Regnault, Fiona Achcar, Isabel M. Vincent, Carl S. Goodyear, Michael P. Barrett

**Affiliations:** 1 Wellcome Centre for Molecular Parasitology, Institute of Infection, Immunity and Inflammation, College of Medical, Veterinary and Life Sciences, University of Glasgow, Glasgow, United Kingdom; 2 Centre of Immunobiology, Institute of Infection, Immunity and Inflammation, College of Medical, Veterinary and Life Sciences, University of Glasgow, Glasgow, United Kingdom; 3 Glasgow Polyomics, Wolfson Wohl Cancer Research Centre, College of Medical, Veterinary and Life Sciences, University of Glasgow, Glasgow, United Kingdom; University of South Alabama Mitchell Cancer Institute, UNITED STATES

## Abstract

Priming and activating immune stimuli have profound effects on macrophages, however, studies generally evaluate stimuli in isolation rather than in combination. In this study we have investigated the effects of pro-inflammatory and anti-inflammatory stimuli either alone or in combination on macrophage metabolism. These stimuli include host factors such as IFNγ and ovalbumin-immunoglobulin immune complexes, or pathogen factors such as LPS. Untargeted LC-MS based metabolomics provided an in-depth profile of the macrophage metabolome, and revealed specific changes in metabolite abundance upon either individual stimuli or combined stimuli. Here, by factoring in an interaction term in the linear model, we define the metabolome interactome. This approach allowed us to determine whether stimuli interact in a synergistic or antagonistic manner. In conclusion this study demonstrates a robust approach to interrogate immune-metabolism, especially systems that model host-pathogen interactions.

## Introduction

Immuno-metabolism is a rapidly growing area of research. Recent studies have shown how metabolites such as succinate and itaconate modulate the function of key innate immune cells such as macrophages [1–4]. Normally, the exposure of macrophages to various stimuli such as cytokines or pathogenic antigens results in the initiation of various signalling cascades through their specific receptors (i.e., cytokine receptors or pattern recognition receptors (PRRs)). Importantly, metabolites act not only as precursors for anabolic and catabolic processes but engage with these intracellular and extracellular signalling pathways to alter the cell phenotype drastically. Perturbations in pathways such as glycolysis and the pentose phosphate pathway (PPP) are required to meet the inflammatory cells’ demands for ATP, NADPH, and ribonucleotide precursors.

Inflammatory macrophage upregulate the production of metabolites such as succinate and itaconate, which have key effector functions. In macrophages, itaconate is produced from citrate that accumulates as a result of the downregulation of isocitrate dehydrogenase [2]. Itaconate has recently been proposed to limit inflammation by inhibiting succinate dehydrogenase, leading to an accumulation of succinate [3,4] although other possible mechanisms, including itaconate acting as a trap for coA [5] and thus affecting macrophage metabolism directly have also been suggested. Moreover, this metabolite can also act as an inhibitor of microbial isocitrate lyase, which was proposed to offer a direct bacteriocidal effect [6].

In contrast to inflammatory macrophages, anti-inflammatory macrophages rely on mitochondrial oxidative phosphorylation with an intact TCA cycle that is supplemented by glutamine. Certain key metabolic processes are differentially regulated in anti-inflammatory macrophages. For example, they increase uptake of triglycerides via the CD36 receptor for use in fatty acid oxidation [7]. Recently it has been found that pathogen generated substrates such as butyrate or indolepyruvate promote an anti-inflammatory phenotype [8,9]. In the context of immune-tumour interactions, tumour associated macrophages reduce glucose availability and promote the formation of an organized tumor vasculature [10].

To generate inflammatory macrophages for metabolic profiling, different groups have used different stimuli. For example, Tannahill *et al* used LPS [1] while Jha *et al* used IFNγ + LPS [2]. These studies have not, however, distinguished between what these stimuli do on their own and what they do together. An obvious example is the argino-succinate shunt, which Jha *et al* demonstrated was upregulated in an attempt to replenish a fragmented TCA cycle. An essential part of this shunt is L-citrulline generated by iNOS (inducible Nitric Oxide Synthase), an enzyme that is maximally upregulated in macrophages treated with both IFNγ + LPS. In the context of anti-inflammatory macrophages, IL-4 is commonly used. While these cells are primed towards an anti-inflammatory phenotype, the effects of pathogen-related stimuli e.g. LPS, have not been explored at a metabolic level.

To address this gap in knowledge, with regard to the impact of inflammatory- and anti-inflammatory-driving stimuli on macrophage metabolism, studies were undertaken to investigate mono- vs combination-stimuli. To model inflammatory macrophages, naive cells (M0) were stimulated with either IFNγ (primed cells), LPS or both IFNγ (primed) and LPS. The rationale behind this was to systematically determine the immuno-metabolic processes that were driven by the immune system (IFNγ), a pathogen (LPS) or the interaction of both (IFNγ + LPS). Similarly, to model anti-inflammatory macrophages, ovalbumin-immunoglobulin immune complexes (OIC), LPS, and OIC plus LPS were compared with M0 macrophage.

## Materials and methods

### Reagents

All cell culture media, serum, and supplements were purchased from Gibco^®^. The stimuli used were murine IFNγ (Peprotech, 315–05), LPS (Sigma: *E*. *coli* 0111:B4, Ref L2630) and murine IL-4 (Ebioscience: 14–8041). IFNγ (100 U/mL: 0.02 μG/mL) was typically added where indicated for overnight stimulation, after which either LPS (100 nG/mL) or OIC were added for indicated times. To make OIC, 136.4 μg anti ova (Creative diagnostics: DPAB26522 polyclonal rabbit serpinb14)) and 13.63 μg albumin (Sigma: A3912) were dissolved in dPBS (Gibco, Magnesium and Calcium free), mixed and incubated at 37°C for 30 minutes prior to use. If different concentrations were used, these are indicated. Note that LPS and OIC were added to wells without replacing medium (i.e. IFNγ was not removed).

### Animal procedures: Macrophage generation

C57BL/6 mice (8–12 weeks old) were bred and housed under standard laboratory conditions at the University of Glasgow (Glasgow, Scotland). All experiments were performed under UK Home Office License. Mice were culled by a Schedule One method (exposure to carbon dioxide gas in a rising concentration) that is authorized by the Animals (Scientific Procedures) Act 1986. To generate bone marrow derived macrophages (BMDM), bones were harvested and cut at each end, and a 23-gauge needle (Kenke Sass Wolf Fine-Ject^®^) was used to flush out bone marrow with complete RPMI (RPMI (Thermo Fisher) supplemented with 2 mM L-glutamine, 1% (v/v) penicillin streptomycin solution (Sigma), 10% FBS (Thermo Fisher) :cRPMI) into a 9 cm Petri dish (Thermo Fisher). To obtain a single cell suspension, cells were passed through a 70 μM cell strainer (Easy strainer^™^ Greiner bio-one), pelleted by centrifugation (300 RCF, 5 min) and resuspended in cRPMI to obtain a density of 6–7 x 10^6^ cells/mL. Cell number was calculated using Trypan blue exclusion (1:1 cells: Trypan blue (Sigma)) to ensure that >95% were viable. The BMDM were matured using 20% L929 supernatant over 7 days (5% CO2, 37°C). In order to quantify M-CSF levels, a Mouse M-CSF DuoSet (R&D Systems) was used with L929 supernatant, according to manufacturer’s instructions. The amount of M-CSF in the L929 supernatant ranged between 165 pg/mL to 285 pg/mL (S1 Fig). Thus, depending on the batch used the cultures will be receiving 33–57 pg/mL; less than a half log difference.

On day three, 5 mL cRPMI and 2 mL L929 supernatant were added. On day six, BMDM were harvested. Medium was removed by aspiration and plates were washed once with warm dPBS to remove remaining non-adherent cells. Note that this wash was only done for experiments involving overnight IFNγ treatments. To each plate 6 mL of ice-cold dPBS was added for 1–2 minutes. Cells were detached by gentle scraping, transferred to a 50 mL falcon tube. Plates rinsed once more with 6 mL of ice-cold dPBS, transferring the same 6 mL between plates to collect remaining cells. Cells were pelleted by centrifugation (300 RCF, 5 min), resuspended in cRPMI and viable cell number was calculated using Trypan blue exclusion. Cell density was then adjusted by dilution to the desired density (10^6^ cells/mL in this study). Next, cells were left to re-adhere overnight in tissue culture plates. Typically 6, and 96 flat well plates were used (Costar).

### Characterisation of BMDM

Flow cytometry analysis was used to verify that BMDM had expected phenotypic surface markers (S2 Fig). For cell surface staining, 10^6^ cells were detached from plates as described above, transferred to 5 ml FACS tubes (BD Falcon), washed twice with 1 mL of dPBS (Mg^2+^ and Cl^-^ free, 1 mM EDTA). Viability staining was executed using APC-eFluor 670 (Ebioscience) according to manufacturer’s instructions. This protocol ends with cells in FACs buffer (PBS, 1 mM EDTA, 2% FBS). For these experiments, alongside fluorescent–1 (FLO-1) controls, compensation beads (eBioscience: OneComp eBeads) were used with each antibody (Biolegend: Cd11b [Brilliant violet 510, Rat IgG2a, κ] and F4/80 [Brilliant violet 421, Rat IgG2a, κ]), according to manufacturer’s instructions. For nitrite determination, supernatants were transferred to a 96 well plate, centrifuged (300 RCF, 5 minutes) and 50 μL transferred to a new 96 well plate. Standard curves were made using a serial dilution from 0.1 M Nitrite (Sigma). The concentrations used were 100, 50, 25, 12.5, 6.25, 3.125 and 0 μM. To the samples and standards, 100 μL of Greiss reagent (Sigma: 03553) was added and plates read at 570 nm using a FLUOstar OPTIMA micro-plate reader (BMG Labtech). Medium incubated in empty wells were used as matrix blanks.

### Sample preparation for LCMS

**Fig 1:**
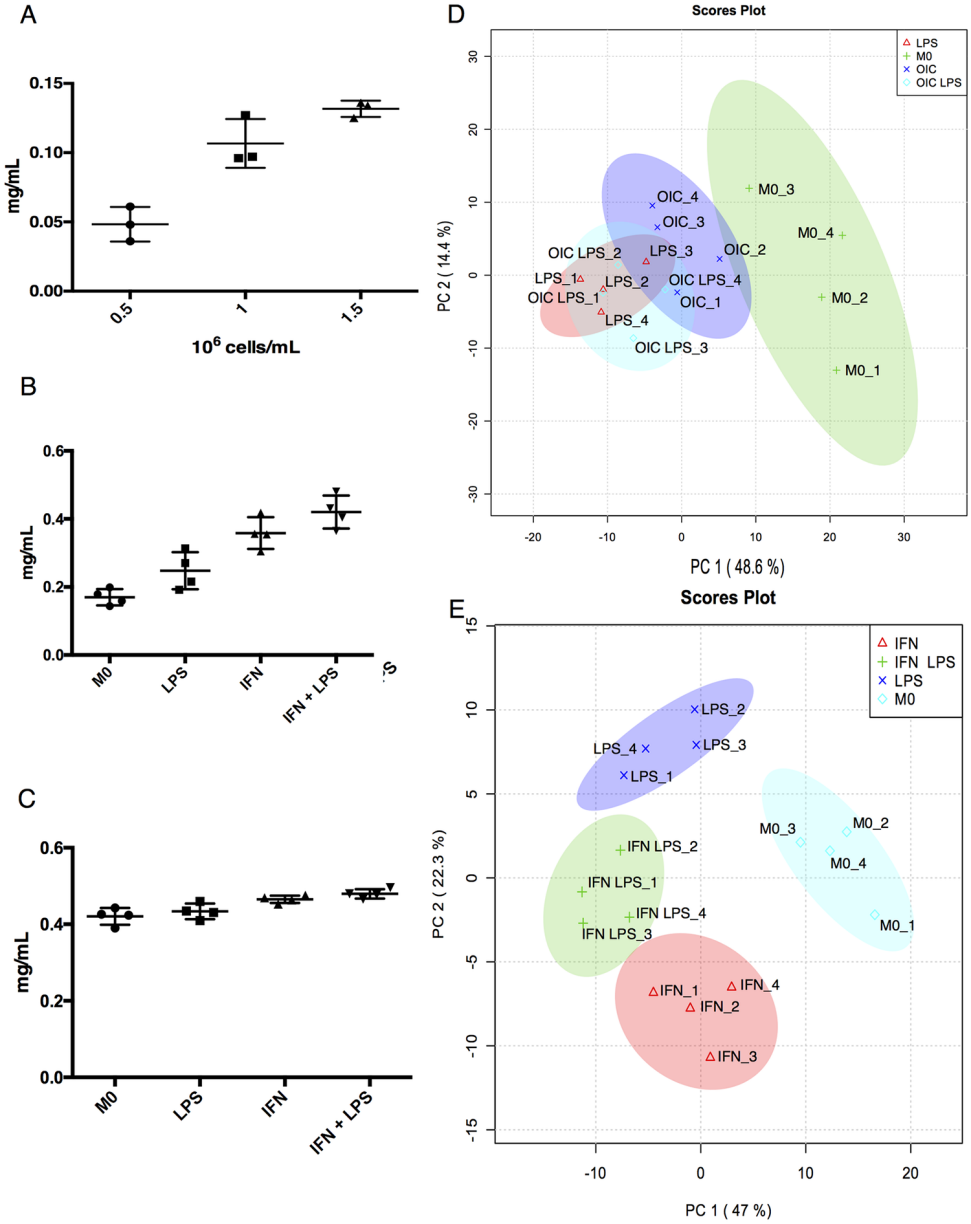
Method of quantifying protein content (mg/mL) post extraction. (A): Macrophages were seeded at densities indicated (n = 4) and protein content quantified post extraction. Results are representative of two independent experiments. (B): The same protocol was employed post extraction on cells cultured using a standard protocol (n = 4). Two sample groups were primed with IFNγ (100 U/mL) overnight, then LPS (100 ng/mL) for 4h (IFN LPS) while the other sample did not get LPS (IFN). One group of samples was treated with LPS without prior IFNγ stimulation (LPS) while M0 is non-treated. The cells that did not receive a given stimulus received an equal volume of cRPMI. (C) As in B, protein quantification was carried out post extraction on samples (n = 4) that were prepared using the modified culturing protocol. Stimuli use and duration were as used in B. (D) PCA on anti-inflammatory (Dataset 1, n = 4) and (E) inflammatory (Dataset 2, n = 4) macrophage data. For D, either LPS (100 nG/mL), ovalbumin-immunoglobulin immune complexes (OIC) (136.4 μg anti ova:13.63 μg albumin), or both were added for six hours. For E, culture conditions and stimuli treatment were as used in C. Note that C is the actual protein measurements of the samples in E. Data was log transformed prior to analysis. MetaboAnalyst 3.0, which utilises the pcaMethods Bioconducter package [17] was used to construct PCA plots. 95% confidence intervals are highlighted by the respective background colour.

**Fig 2:**
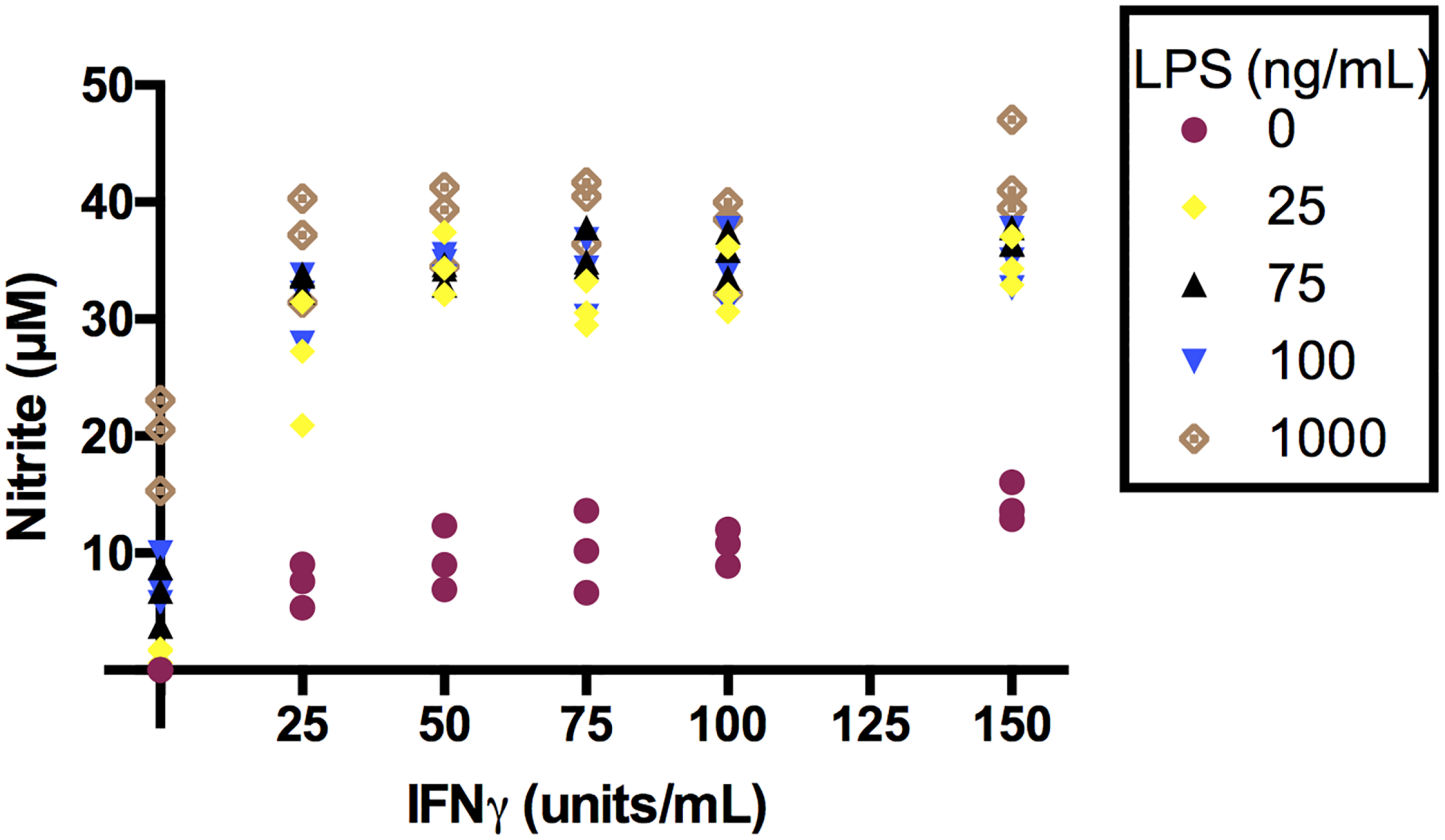
Response in nitrite levels to varying stimuli levels. Cells were primed with IFNγ (indicated concentrations) overnight, and subsequently with LPS (indicated concentrations) for 24h. Nitrite was quantified using the Griess assay (n = 3).

**Fig 3:**
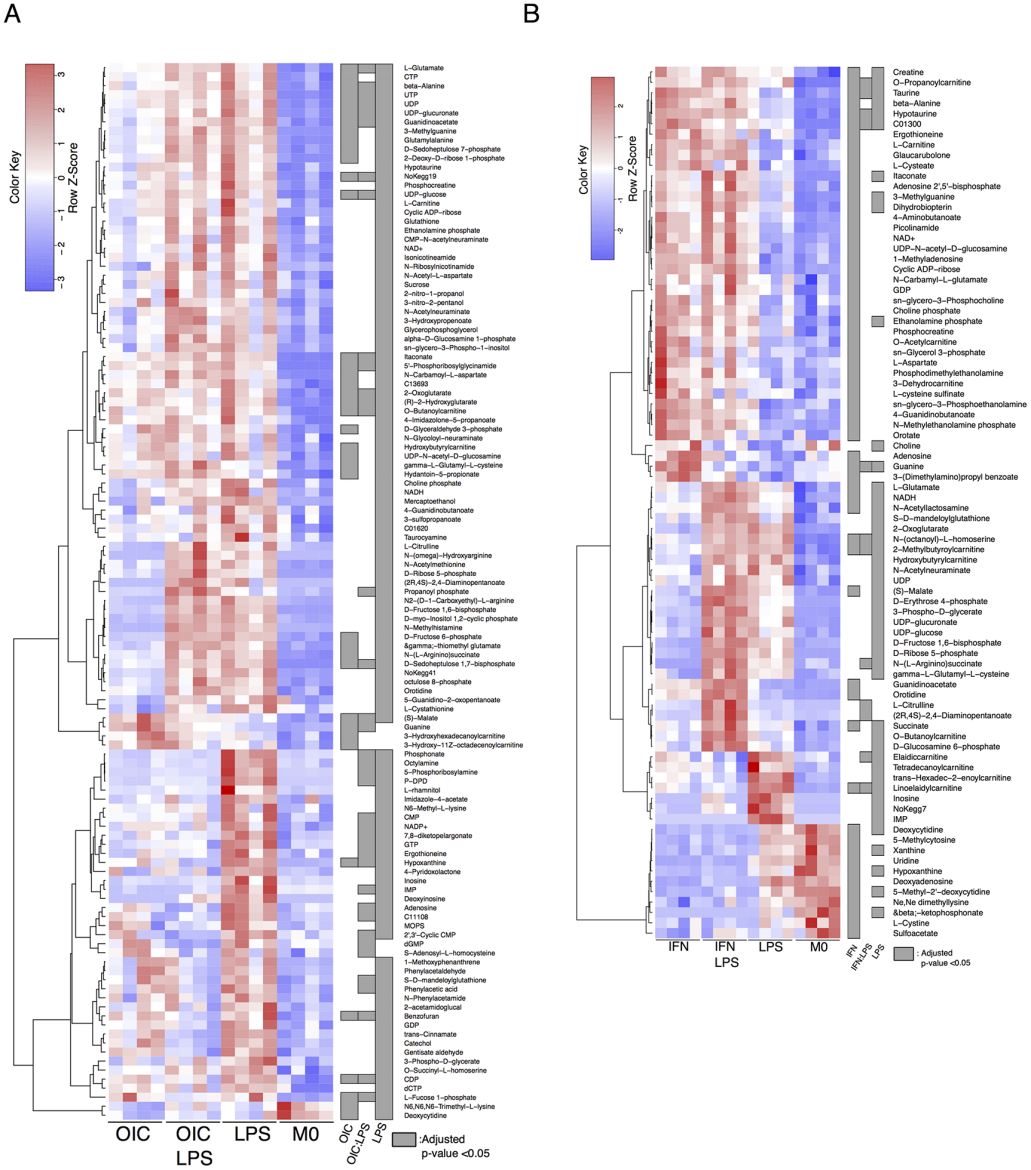
Heatmaps showing metabolites with significantly (GLM FDR < 0.05) altered levels. Treatment groups are as indicated (n = 4 for both 3a and 3b). For the “Significance” grey refers to GLM corrected p-value (FDR< 0.05). Dataset 1 (anti-inflammatory, Fig 1D) is shown in the first heatmap and Dataset 2 (inflammatory set, Fig 1E) in the second.

**Fig 4:**
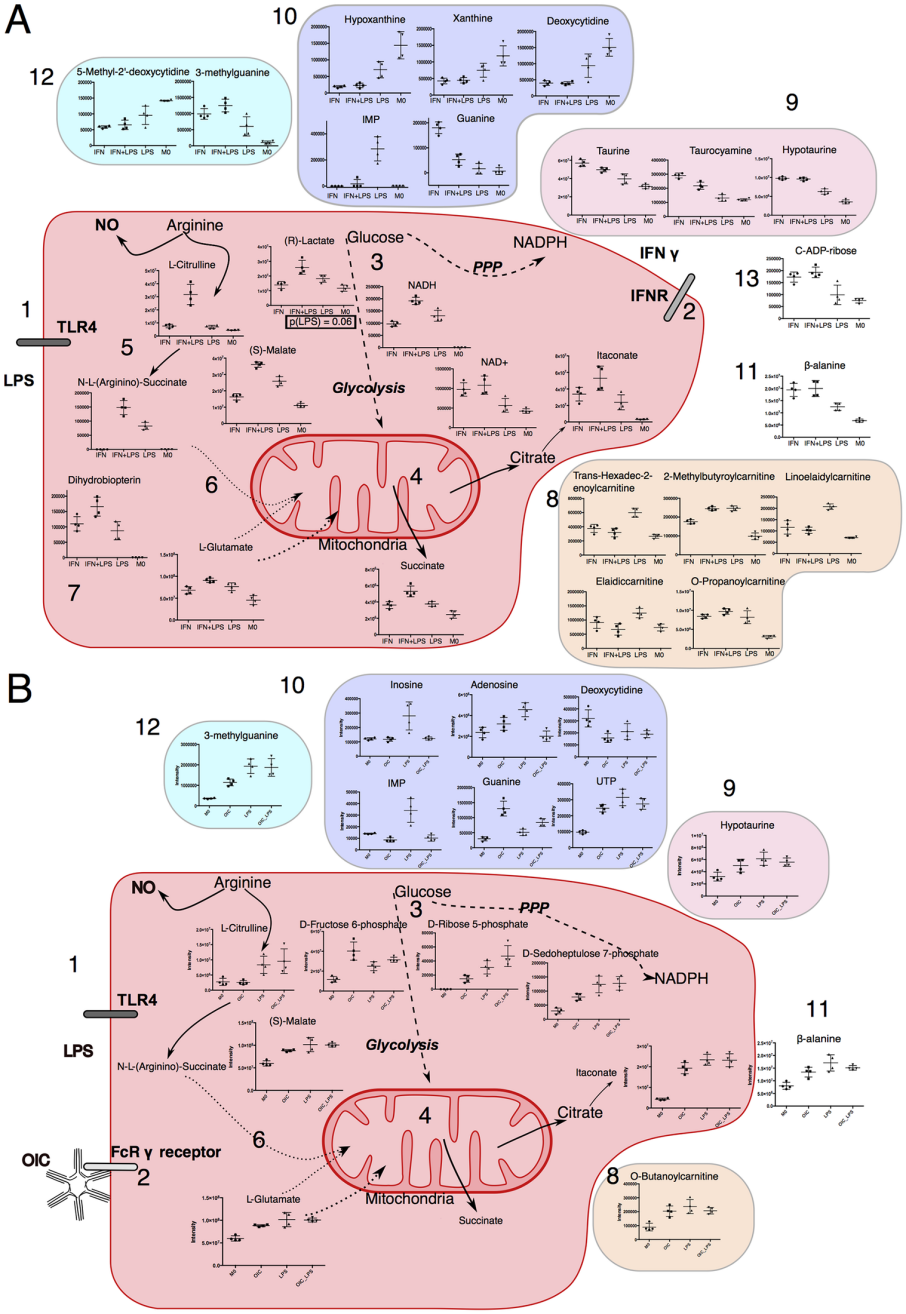
Our results on (A) inflammatory macrophages and (B) anti-inflammatory macrophages in the context of macrophage immune-metabolism. Note that location of metabolites does not refer to cellular location. 1: & 2: Stimuli (LPS and IFNγ) were used as described in Fig 1. 3: This causes increase in metabolites in glycolysis ((R))-Lactate) and the PPP (S4 File). 4: Previously reported perturbations in the TCA cycle are indicated including Itaconate production and Succinate accumulation. 5: L-Arginine is metabolised to L-Citrulline and effector molecule NO*. 6: Arginine and Glutamate metabolism are connected with N-L- (Arginino)-Succinate and aspartate (from glutamate) allow for replenishment of the TCA cycle at the point of Fumarate. 7: Dihydrobiopterin is used to form the iNOS cofactor tetrahydrobiopterin by Dihydrobiopterin reductase. 8: Levels of select carnitine related metabolites are modulated by LPS and/or IFNγ. 9: Taurine metabolism is modulated by IFNγ. 10: Purine and pyrimidine metabolism displays as stimuli specific profile as did some modified analogues. 11: Levels of β-alanine, which can originate from arginine, pyrimidine fatty acid or glutamate metabolism, were increased in inflammatory macrophage. 12: alterations in nucleotide analogues were present in both datasets. 13: C-ADP-ribose in increased in an IFNγ dependent manner. For colour schemes, blue refers to nucleotide analogues, purple to purine and pyrimidine metabolism; orange to carnitine related metabolites; red refers to central carbon and arginine metabolism; and pink refers to taurine metabolism. Differences in metabolites present in each A and B is due to a lack of significant changes or metabolite not being detected. Direct reactions are in bold lines; dashed lines show multistep reactions.

3 million macrophages/well/replicate were seeded in a 6 well plate at a density of 10^6^ cells/mL and extracted in a volume of 400 μL chloroform/methanol/water, 1:3:1 (CMW). Medium was aspirated and cells were washed once with dPBS (Magnesium and Calcium free, no EDTA), aspirating immediately. Ice-cold extraction solvent (chloroform/methanol/water, 1:3:1) was added to each well, including an empty well (for solvent blank), and cells were left shaking (1 hour, 4°C). Samples/replicates were not pooled. Solvent was then transferred to 1.5 mL micro-tubes, and centrifuged (18,000 RCF, 15 minutes, 4°C). Supernatant was immediately transferred to 2 mL screw-cap tubes. Sample from each tube was transferred to a new screw-cap tube for a pooled sample used in mass spectrometry quality control. At this point, samples were capped with Argon, and then stored at -80°C until LC-MS analysis. To calculate protein levels post extraction, NaOH (0.1 M, 400 μL/10^6^ cells) was added to each well. The plates were left shaking (15 minutes 4°C). Plates were next centrifuged (300 RCF, 5 min) and 10 μL of supernatant transferred to a 96 well plate. Note that for each replicate well (n = 4) 3 (Fig 1a) -4 (Fig 1b) technical replicates were taken for protein concentration measurement. To the same plate, a 10 μL of a BSA (Sigma) dilution series (2 fold: 0.5–0 mg/mL in 0.1 M NaOH) was added to allow for determining of protein concentration. Finally, 190 μL of Bradford reagent (BioRad) was added to each plate on top of samples and standards. Absorbance was measured at 595 nm using a FLUOstar OPTIMA micro-plate reader. In Fig 1a and 1b the average of the technical replicates is shown.

### LCMS

All samples were separated with high performance liquid chromatography (HPLC) on a Dionex Ultimate 3000 RSLC system (Thermo) using ZIC-pHILIC (Merck) column. The mass spectrometry platform used was a qExactive Orbitrap mass spectrometer (Thermo). Analysis was performed in positive and negative mode; using 10 μL injection volume and samples were maintained at 4°C during analysis. A linear biphasic LC gradient was conducted from 80% B to 20% B over 26 minutes, where solvent B was acetonitrile and solvent A was 20 mM ammonium carbonate in water. The flow rate was 300 μL/min, and column temperature maintained at 25°C. For longer LC protocol the gradient was identical except that it was over 46 minutes. Each sample was run in a randomized order, with a pooled sample run between every 4 samples.

The MS set up was calibrated [Thermo calmix (Pierce^™^ calibration solutions from Thermo Scientific) with masses at lower m/z; 74.0393 m/z (C2H6NO2: +) and 89.0244 (C3H5NO3: −)] in both ionization modes before analysis and a tune file targeted towards the lower m/z range was used. Full scan (MS1) data was acquired in both ionization modes in profile mode at 50,000 resolution (at m/z range 70–1400), an automatic gain control (AGC) target of 106 cts, with spray voltages +4.5 kV (capillary +50 V, tube: +70 kV, skimmer: +20 V) and −3.5 kV (capillary -50 V, tube: -70 kV, skimmer: -20 V), capillary temperature 275°C, probe temperature 150°C, sheath gas flow rate 40, auxiliary gas flow rate 5 a.u., and a sweep gas flow rate of 1 a.u. For fragmentation, the settings are in S1 File and the analysis was conducted using mZCloud.

### Data processing and analysis

Data analysis was performed using the XCMS [11] mzMatch [12] and IDEOM [13] software for untargeted analysis. Xcalibur (Thermo) was used for targeted peak picking and exporting fragmentation spectra, which were used as queries with which to search mzCloud [14]. A mixture of 240 standards (in three separate mixes), covering a range of metabolic pathways, was run alongside each sample batch to allow metabolite identifications (MSI level 1). According to the metabolomics standards initiative (MSI), metabolite identifications (MSI level 1) are given when more than one feature matches an authentic standard (i.e., mass and retention time) while annotations are made when matching to a metabolite is made by mass only (MSI level 2) [15].

Peaks were visually interrogated on the identification tab of the IDEOM spreadsheet, resulting in some annotated features being removed at this stage due to poor peak quality. To avoid the lipid bolus, a 4.2-minute retention time cut off was applied. As lipids and peptides are not reproducibly detected using this platform, they were removed from statistical analysis. If available, fragmentation data was used to strengthen confidence in identifications. PLSDA statistical analysis was conducted using MetaboAnalyst on log-transformed data. To avoid over-fitting, 1,000 permutations were run using prediction accuracy during training as well as separation distance (the ratio of the between sum of the squares and the within sum of squares: B/W). Graphpad Prism (One way ANOVA with Tukey’s correction for pairwise comparisons) was used to test variance between batches of MCSF levels in L929 supernatant and significance denoted as adjusted p less than or equal to 0.05). For the GLM analysis conducted using R [16] with the Benjamini-Hochberg procedure (false discovery rate (FDR) less than 0.05). Finally, β-alanine and L-alanine could be separated based on retention time but the gap was too small for standard settings within the IDEOM pipeline [13]. For these metabolites, a custom method was written in Xcalibur (Thermo) where an appropriate retention time window (±30 seconds of authentic standard) was used to obtain an accurate peak area measurement.

## Results & discussion

### Standardising sample preparation and global metabolic profiling

Overnight priming with IFNγ led to increased cell density with fewer non-adherent cells. Therefore, to ensure that samples were comparable, we quantified the levels of protein that was precipitated during the extraction protocol (Fig 1A). This revealed that IFNγ treated cells had higher protein levels (Fig 1B) so our culturing technique was modified to account for this (Fig 1C). Here we added a washing stage with warm (37°C) PBS to ensure that macrophage subsequently removed via scraping for use in experiments were firmly attached. In both experiments, using different types of inflammatory or anti-inflammatory macrophage, clear separation was evident when the data was subjected to principal component analysis (PCA) with the inflammatory macrophages being the most distinct (Fig 1D and 1E). For the inflammatory macrophage dataset, filtering in IDEOM and the Xcalibur software resulted in a list of 233 metabolites. The identity of 74 of these was confirmed using authentic standards (MSI level 1). For the anti-inflammatory macrophage dataset, 372 metabolites were annotated, with 98 of these confirmed using authentic standards (MSI level 1).

We conducted Partial Least Squares Discriminant Analysis (PLSDA) to obtain VIP scores (a measure of a variable's importance) for metabolites in both data sets. Similar to the results from PCA, there was separation between sample classes in both datasets (S3 Fig). PLSDA is a supervised method (not blind to class types) and it can over-fit data. To decrease the possibility of this occurring, 1,000 permutations were run using prediction accuracy during training as well as separation distance (the ratio of the between sum of the squares and the within sum of squares: B/W). For both datasets, results of both permutation tests each gave a p value <0.02, hence the method was applied herein.

There was some overlap between both datasets in the top 80 results (VIP score). Metabolites of interest included IMP, inosine, guanine, D-ribose 5-phosphate, adenosine and itaconate. The complete results from this analysis are located in S2 File. While these metabolites belong to pathways that are known to be important for macrophage function, this approach did not take into account the extent to which each immune stimulus contributed to perturbations in these pathways. For inflammatory macrophages, iNOS activity is a key part of their microbicidal machinery. The activity of this enzyme is maximised in the presence of both IFNγ and LPS (nitrite levels shown in Fig 2) so this would be an example of a metabolite that could be subject to an interaction effect. Note that Fig 2 is a separate experiment. As L-citrulline is a by-product of this enzyme and given that it has recently been demonstrated that this metabolite is required to supplement the anaplerotic TCA cycle present in inflammatory macrophage, we chose a method of statistical analysis that allows testing for interactions between the various immune stimuli used.

### A generalised linear model, categorising interactions

For analysing both datasets a generalised linear model (GLM), as implemented in the R coding language (GLM) (S3 File), was used. GLM is a generalised instance of the linear model LM (e.g., ANOVA procedure) [16]. Here the permutations were performed using a least squares regression approach to describe the statistical relationship between one or more predictors (in this case the stimuli) and a continuous response variable (in this instance a given metabolite intensity).

Since we were interested in assessing whether one immune stimulus could affect another we incorporated an interaction term to determine whether two predictor variables affect the outcome variable in a way that is non-additive. This is particularly appropriate for this experiment as it is widely accepted that the combined effect of IFNγ and LPS can increase inflammation (e.g., Fig 2). In general terms as implemented here:

model=glm(Y~X1+X2+X1:X1) (1)

In R, this function regresses Y on X1 (IFNγ or OIC), X2 (LPS), and the X1-by-X2 (IFNγ or OIC-by-LPS) interaction term.

The input file required for this analysis in text format and the results of this analysis are in S4 and S5 Files. A list of all metabolites detected in both experiments, confirmation of identification using accurate Mass (MS1), authentic standards or fragmentation can be found in S6 File.

**S**timuli specific signatures induced by OIC/IFNγ and/or LPS (Fig 3) denotes effects that are additive, antagonistic or synergistic; with increases or decreases in metabolite levels noted in comparison to the non-treated control (M0). An example of this can be seen in Dataset 2 (inflammatory set) where IFNγ and LPS each perturb the levels of L-citrulline on their own but in combination are highly synergistic.

#### IFNγ, a primer and active agent

In Dataset 2 (inflammatory set), IFNγ causes a clear depletion of purine and pyrimidine related metabolites such as xanthine and uridine, and in some cases, this is enhanced by the presence of LPS (e.g. hypoxanthine). In purine metabolism, xanthine oxidase (XO) catalyses the conversion of hypoxanthine to xanthine, producing H_2_O_2_ as a by-product. The H_2_O_2_ produced by XO has been shown to activate the p38-MAPK-NFAT5 pathway and inhibiting XO with allopurinol, limited inflammation in a mouse model of arthritis (14). Perturbations to levels of both the substrate and product of XO in stimulated cells were also observed. It is notable that increases in many of the hallmark metabolites of immune-metabolism can be explained by only one stimulus or a slight additive effect. For example, metabolites involved in glycolysis (fructose 1,6-bisphosphate and 3-phospho D-glycerate), the TCA cycle (2-oxoglutarate), and the PPP (ribose 5-phosphate and erythrose 4-phosphate), are all primarily driven by LPS.

Both LPS and IFNγ induce several classical inflammatory metabolites such as succinate, L-glutamate and (S)-malate. Both these stimuli also increased itaconate levels, although this metabolite has recently been proposed to limit inflammation [3,4], suggesting that its elevation may act as a constraint to macrophage activation. Perturbations were also detected in NAD^+^ and NADH levels, which have roles in the cells’ redox state (Fig 4). Note that the LC-MS platform employed in these studies is not optimised to detect metabolites in their native redox state. Glutathione is critical to cellular redox chemistry. Significant differences in either reduced (GSH), or oxidised (GSSG) were not apparent. It is important that the roles of the single treatments and the combinations used here be investigated in the context of cell redox state using biochemical assays designed specifically for these metabolites.

The increase of several acylcarnitines by LPS e.g. trans-hexadec-2-enoylcarnitine, elaidiccarnitine and linoelaidylcarnitine is striking. Notably, elaidiccarnitine and linoelaidylcarnitine had similar retention times and thus one may be an adduct of the other. Adding IFNγ with LPS, however, reverses the stimulatory effect of LPS on these metabolites. L-carnitine and other O-acetyl-L-carnitine are, however, increased in the presence of IFNγ with the presence of LPS having little effect. Importantly, an increase in L-carnitine levels has previously been observed in anti-inflammatory macrophage [2]. Further work will be needed to elucidate whether these metabolites have a function in a pro-/anti-inflammatory (or both) responses in the context of macrophage metabolism. This is especially relevant when considering that macrophage activation has been shown to be a spectrum rather than a binary pro/anti-inflammatory system at a transcriptional level [18].

Inosine monophosphate (IMP) displays a similar trend to trans-hexadec-2-enoylcarnitine, elaidiccarnitine and linoelaidylcarnitine in that it was increased in LPS treated samples but not in samples treated with both IFNγ and LPS (S4 Fig). Similarly, inosine was increased in LPS treated samples but while it was lower in the combination treatment, this did not reach statistical significance.

#### Possible role of OIC in counteracting effects of LPS

While there are some similarities to the role of OIC and IFNγ, it is clear that LPS is the dominant stimulus in Dataset 1 (anti-inflammatory set), although the variability within groups visible in Fig 3 may contribute to this. Pyrimidines, purines, L-carnitine, hydroxybutyrylcarnitine and O-butanolycarnitine (the latter two are acyl-carnitines) are mostly increased when compared to the M0 group. Two acylcarnitines are increased in an OIC dependent manner. The acylcarnitines measured here reveal complex patterns with regard to increases and decreases with various stimuli. Ascertaining specific roles for these metabolites will be an intriguing subject of future studies.

Both OIC and LPS cause an increase in itaconate, which has been shown to limit inflammation. As this is the first study to use OIC to interrogate immune metabolism, further studies will be required to elucidate the mechanisms and consequences of this increase in itaconate. Itaconate appears to have complex roles in inflammation. For example, it has been proposed to have direct anti-bacterial properties [6], but also dampen inflammatory response [3,4]. The results reported here conform to this complex role of itaconate in the inflammatory response. L−citrulline, N−(L−arginino)succinate, and D−fructose 6−phosphate and are all increased by LPS and these inflammatory signature metabolites remain increased even when OIC are present. Note that these increases driven by LPS match that of previous studies that examined metabolism [1] or metabolism and transcriptomics [2] of inflammatory macrophages but here the effect of LPS and IFNγ can be separated. (S)-malate and guanine are both increased by OIC, an effect which is abrogated by the presence of LPS. Interestingly, in both datasets, the priming stimuli on its own caused a slight increase in guanine, which was further increased in the presence of LPS. The converse of this was true for hypoxanthine and IMP, which decrease when both stimuli are present (S4A and S6A Figs).

This may be a consequence of increased IMPDH activity (which catalyses the conversion of IMP to xanthosine monophosphate (XMP, not detected), as the first committed step towards the *de novo* biosynthesis of guanine nucleotides (S4A and S6B Figs). Increases in the abundance of transcripts from genes encoding the guanosine metabolising enzymes pnp and pnp2, which also catalyse inosine-hypoxanthine interconversions has also been noted [2]. A key immune-modulatory role for IMPDH has been proposed [19]. In that study, the authors found that the IMPDH inhibitor mycophenolate mofetil suppressed production of pro-inflammatory cytokines, nitric oxide, and lactate dehydrogenase in a macrophage cell line. It is important to note that in these experiments the intensity of IMP and inosine was near the lower detection limit of our LCMS system. Interestingly, adenosine, which is known to down-regulate classical macrophage activation [20], is increased in an IFNγ-dependent manner. In Dataset 1 (anti- inflammatory set), the increase in adenosine induced by LPS is repressed by the presence of OIC. Whether this is indicative of a regulatory mechanism remains to be determined. Finally, there were two modified nucleotide analogues that had altered levels; 5’-methyl-2’deoxycytidine (marker of *de-novo* DNA methylation: decreased by all stimuli) and 3-methyguanine (altered in leukaemia, tumours and immunodeficiency: increased by all stimuli).

Thus, perturbations in these pathways are not explained by just one stimulus simply increasing or decreasing overall levels. Nucleotides clearly have important roles in cellular signalling pathways as well as nucleic acid biosynthesis and untangling the varied contributions of purine metabolism to macrophage differentiation will be a key challenge in understanding immunometabolism more generally. Metabolic responses to different stimuli can be different, contradictory and certainly complex. This implies that studies involving single or even a limited set of stimuli will not create a true reflection on the complicated picture *in vivo*.

### Pathway analysis

To formally categorise the contribution of LPS and OIC or IFNγ to biological pathways, pathway analysis was performed on log-transformed data using the pathway analysis module in MetaboAnalyst. This allowed us to use the murine pathway library and upload a relevant background (all detected metabolites). The use of a background is important as it allows technical bias specific to the instrument used to be taken into account.

Herein, a list of all detected metabolites was used as the background. The Pathway Enrichment Analysis used is based on the GlobalTest algorithm [21]. To estimate node importance, the Relative-betweeness Centrality algorithm was selected. This measures the number of shortest paths going through the node, focusing more on global network topology. Thus changes in metabolites at central nodes within or between pathways are given more importance than those at the extremities as they are more likely to effect the pathway (s) [21].

First, metabolites either effected by LPS or IFNγ (OIC in the case of Dataset 1), or the interaction of the two: IFN:LPS (OIC:LPS in the case of Dataset 1) were analysed separately and together (Table 1). An impact score of ≥0.1 was used as a threshold to select a shortlist of pathways for further investigation.

**Table 1:** Pathway analysis of inflammatory (Dataset 2) and anti-inflammatory (Dataset 1) macrophage datasets.

**Dataset 2**													
	Total Compounds	**IFN list**			LPS list			Interaction list			Entire list		
		FDR	Impact	Hits	FDR	Impact	Hits	FDR	Impact	Hits	FDR	Impact	Hits
Pyrimidine metabolism	12	0.003	0.021	2							0.002	0.021	2
Butanoate metabolism	5	0.005	0.029	1							0.000	0.029	2
Citrate cycle (TCA cycle)	8				0.002	0.068	1				0.002	0.068	1
beta-Alanine metabolism	4	0.003	0.444	1	0.002	0.444	1				0.002	0.444	1
Glycerophospholipid metabolism	7	0.005	0.044	1	0.003	0.068	2				0.004	0.068	2
Glycine, serine and threonine metabolism	7	0.007	0.031	2	0.019	0.031	2				0.011	0.031	3
Purine metabolism	15							0.000	0.127	2			
Arginine and proline metabolism	18	0.005	0.047	4	0.002	0.023	1	0.000	0.054	2	0.004	0.047	4
Alanine, aspartate and glutamate metabolism	13	0.005	0.114	1	0.002	0.063	1	0.001	0.022	1	0.000	0.177	2
Taurine and hypotaurine metabolism	4							0.001	0.714	2			
Primary bile acid biosynthesis	1							0.001	0.030	1			
Dataset 1													
	Total Compounds	OIC list			LPS list			Interaction list			Entire list		
		FDR	Impact	Hits	FDR	Impact	Hits	FDR	Impact	Hits	FDR	Impact	Hits
Glycerophospholipid metabolism	6				0.010	0.044	1				0.010	0.044	1
Glutathione metabolism	8				0.010	0.003	1				0.011	0.003	1
Histidine metabolism	11				0.016	0.108	1				0.016	0.108	1
Tyrosine metabolism	5				0.021	0.001	1				0.021	0.001	1
Glycine, serine and threonine metabolism	10	0.002	0.031	1				0.002	0.031	1	0.003	0.031	2
Arginine and proline metabolism	20	0.002	0.023	1	0.006	0.012	2	0.002	0.023	1	0.003	0.035	3
Citrate cycle (TCA cycle)	9	0.002	0.068	1	0.003	0.068	1	0.002	0.068	1	0.003	0.068	1
Alanine, aspartate and glutamate metabolism	12	0.002	0.063	1	0.003	0.063	1	0.002	0.063	1	0.003	0.063	1
Pyrimidine metabolism	20	0.002	0.140	5	0.003	0.147	6	0.002	0.133	4	0.003	0.147	6
beta-Alanine metabolism	5	0.002	0.444	1	0.003	0.444	1	0.002	0.444	1	0.003	0.444	1
Purine metabolism	21							0.009	0.005	1	0.011	0.005	1
Phenylalanine metabolism	5				0.018	0.130	1	0.017	0.130	1	0.018	0.130	1

Analysis was conducted on log-transformed data (Kegg IDS) using MetaboAnalyst. Here the GlobalTest was used in conjunction with the Relative-betweeness Centrality algorithm. For each dataset, a list of all detected metabolites (Kegg IDs) was used as a background. Dataset 1 refers to the anti-inflammatory dataset and Dataset 2 refers to the inflammatory dataset. The maximum importance of each pathway is 1, and the pathway impact is the cumulative proportion from the matched metabolite nodes.

As mentioned above, LPS induced accumulation of inosine and inosine monophosphate (IMP) and an IFNγ mediated increased guanine were among the most significant changes detected in our study (note that OIC had a similar effect on guanine). Pathway analysis confirms the perturbation of pyrimidine metabolism by the stimuli used in this study. Whether these changes in nucleotide metabolism represent a predisposition to the transcriptional changes associated with macrophage activation, or else other nucleotide signalling events is unknown. Purine metabolism is also significantly altered in both datasets.

β-alanine metabolism was significantly altered according to pathway analysis in both datasets. There are only 5 metabolites in this KEGG pathway and while it is part of several other KEGG pathways, it has been reported that β-alanine is a by-product of increased flux through arginine metabolism via carnosine synthase 1 (Carns1) in anti-inflammatory macrophages [2]. Carns1 catalyses the conversion of carnosine (no difference detected) and ADP (not detected) to L-histidine (no difference detected), ATP (no difference detected) as well as β-alanine. An alternative source of β-alanine is pyrimidine metabolism that was subject to stimuli specific perturbations (S5 and S6C Figs). Tracing the fate of arginine in polarised macrophage should help to determine if this is the case,

Several studies have investigated the immune-modulating capability of β-alanine [22]. Prabha *et al*, for example, reported that β-alanine caused down-regulation of lipoprotein lipase (LPL) activity and altered cholesterol metabolism. Harris *et al* found that IFNγ inhibits LPL transcription in macrophages [23]. As the increases presented here were present in all combinations of IFNγ treatment, it would be interesting to determine if β-alanine has a role modulating LPL activity. We also detected IFNγ-mediated increases in taurine and its related metabolites hypotaurine and taurocyamine (S4B and S6C Figs). These metabolites have been shown to modulate cholesterol metabolism and LPL activity [22].

## Conclusion

This study has revealed hitherto unexpected complexity in macrophage response to different immune-stimuli. Further targeted analysis (e.g. through isotope labelling studies) and validation studies (medium deprivation and inhibitors) will enable further dissection of roles played by individual metabolites and their pathways in regulating the behaviour of macrophage. By combining single and combinatorial treatment of macrophage with a linear model that incorporates an interaction term, we are able to denote which key pathways are perturbed by each stimulus and whether they interact in a synergistic or antagonistic manner or not, findings what are likely to be relevant when considering the multiple stimuli present *in vivo* (Fig 4). This will be especially important for future studies when taking in to consideration that the diverse range of different TLR signalling pathways, i.e. TLR2, can and do result in different immune-metabolic programme [24] while use of bacteria, virus or co-infections can model *in vivo* situations where multiple signalling cascades are engaged. Other factors to consider are the tissue of origin of either monocytes or mature macrophage or if the tissue is diseased. For example, mature peritoneal macrophages are distinct metabolically from BM derived macrophage [25]. Additionally the differentiation of BM derived macrophage can be achieved by MCSF or GMCSF, the latter of which generates inflammatory-primed macrophage and it would not be unexpected that this could have an effect on the mature macrophage metabolic phenotype. This knowledge will be essential if deciding to target inflammation via the pathogen, immune system, or both.

## Supporting information

S1 Fig MCS-F of 4 batches of L929 supernatant was quantified using ELISA. A one-way ANOVA with a Tukeys multiple comparison test was used to test for significance (p<0.05).
(TIF)

S2 Fig Flow cytometry characterisation of cultured macrophage. (A) Selecting population based on forward scatter area (FSC-Area) against side scatter area (SSC-Area). (B) Gating on single cells using SSC-Area against side scatter area (SSC-Height). (C) Gating on live cells using gating on cells negative for the viability dye Ef780. (D) Gating on Cd11B+ cells then, (E) characterising presence of F4 80. (F) Fluorescence -1 (FLO-1) control for Cd11b and, (G) F4-80. (H) Histogram overlay of D (blue) and F (red). (I) Histogram overlay of (E) (blue) and (I) (red). Results are representative of 3 biological replicates.
(TIF)

S3 Fig (A) PLS-DA conducted on dataset 1 and 2 (log transformed data). The given conditions are denoted in the inset box. (B) 1000 permutations were run using prediction accuracy during training as well as (C) separation distance (B/W).
(TIF)

S4 Fig (A) Purine and (B) taurine metabolism related metabolites from Dataset 2. Significance as determined by GLM is denoted by asterisk. Broken lines denote multi-step reactions.
(TIF)

S5 Fig Pyrimidine metabolism related metabolites from Dataset 2. Significance as determined by GLM is denoted by asterisk. Broken lines denote multi-step reactions.
(TIF)

S6 Fig (A): Purine, (B): pyrimidine and C: Taurine metabolism related metabolites from Dataset 1. Significance as determined by GLM is denoted by asterisk. Broken lines denote multi-step reactions.
(TIF)

S1 File Fragmentation settings used in creating Dataset 2. (TXT)

S2 File Results of PLDA that was conducted on Datasets 1 and 2. (XLSX)

S3 File R-code used to run GLM. (R)

S4 File Input, summary and supplemental information about results of GLM on Dataset 2. For GLM, p values and Benjamini-Hochberg corrected values are shown. In the ANOVA tab, the given pairwise comparisons that are significantly different at the stated false discovery rate are shown. If a metabolite matched an authentic standard in retention time or its fragmentation on mZCLOUD, this is denoted in the final two columns on the GLM significant tab.
(XLSX)

S5 File Input, summary and supplemental information about results of GLM on Dataset 1. For GLM, p values and Benjamini-Hochberg corrected values are shown. In the ANOVA tab, the given pairwise comparisons that are significantly different at the stated false discovery rate are shown. If a metabolite matched an authentic standard in retention time this is denoted in the final column on the GLM significant tab.
(XLSX)

S6 File Confidence of metabolite identification in two datasets is denoted. Authentic standards were run along side samples. MS2 was carried out on pooled sample (Dataset 2 only).
(XLSX)
