# Supplementary figures and images for: Metabolomic profiling of macrophages determines the discrete metabolomic signature and metabolomic interactome triggered by polarising immune stimuli
Source: PLoS One. 2018 Mar 14;13(3):e0194126. doi: 10.1371/journal.pone.0194126 (PMC5851634; doi:10.1371/journal.pone.0194126)

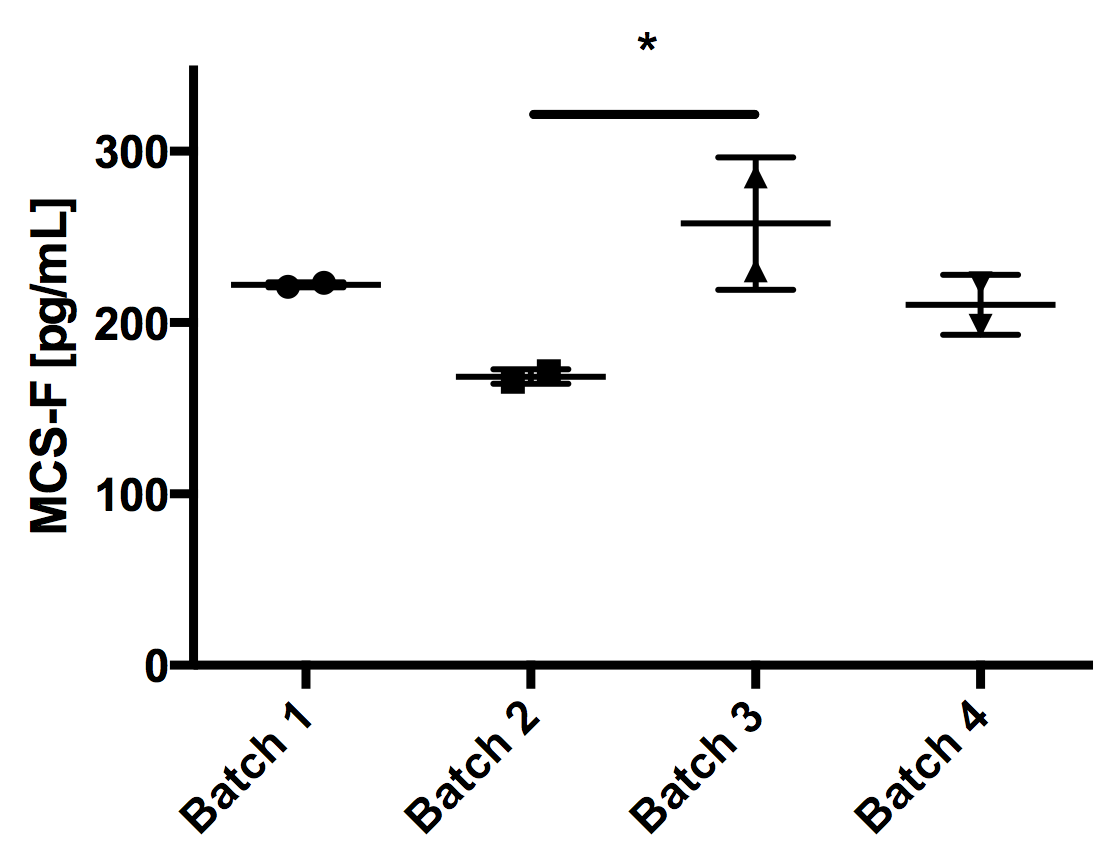

Supplement: S1 Fig — A one-way ANOVA with a Tukeys multiple comparison test was used to test for significance (p<0.05). (TIF) [file pone.0194126.s001.tif]

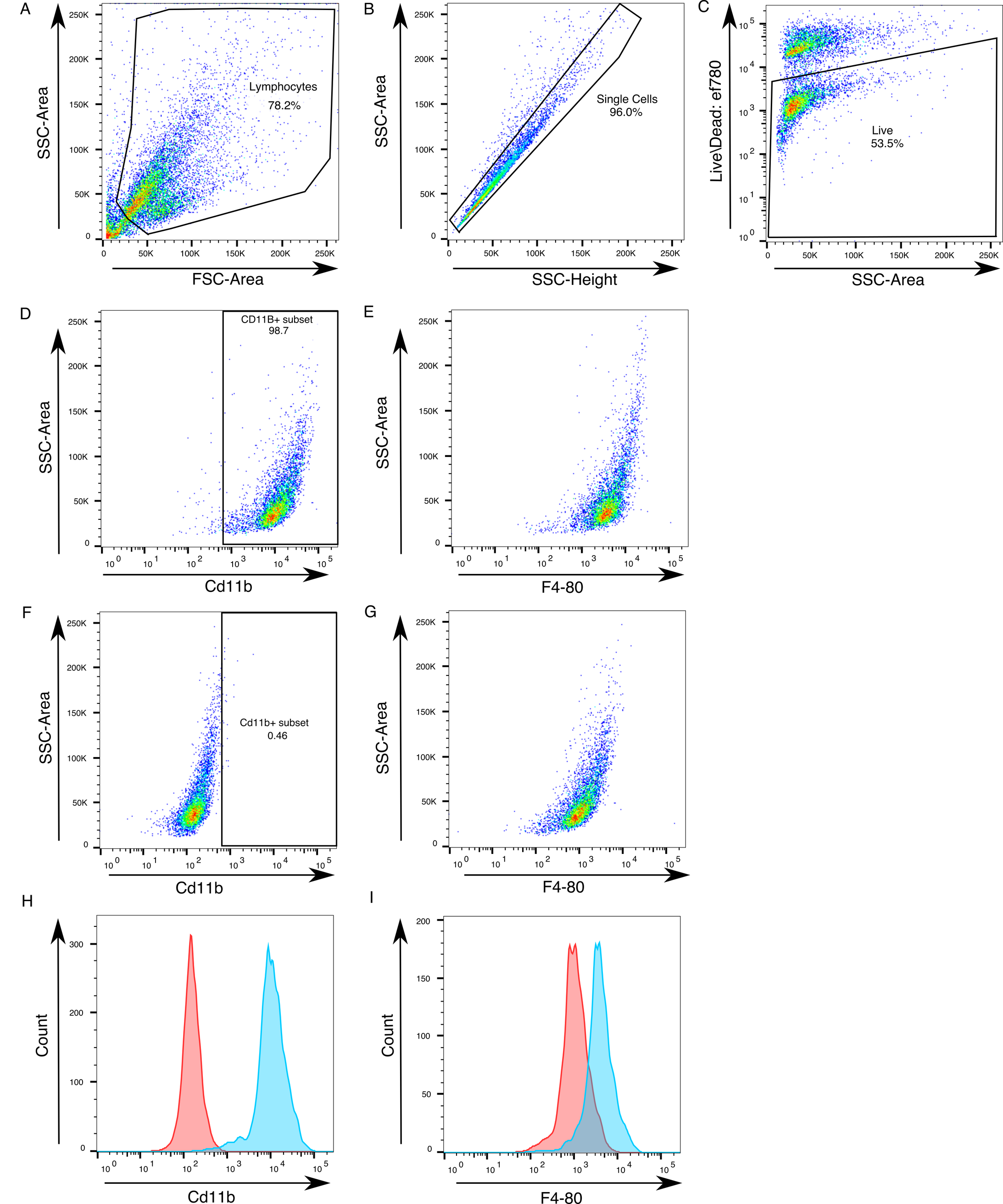

Supplement: S2 Fig — (A) Selecting population based on forward scatter area (FSC-Area) against side scatter area (SSC-Area). (B) Gating on single cells using SSC-Area against side scatter area (SSC-Height). (C) Gating on live cells using gating on cells negative for the viability dye Ef780. (D) Gating on Cd11B+ cells then, (E) characterising presence of F4 80. (F) Fluorescence -1 (FLO-1) control for Cd11b and, (G) F4-80. (H) Histogram overlay of D (blue) and F (red). (I) Histogram overlay of (E) (blue) and (I) (red). Results are representative of 3 biological replicates. (TIF) [file pone.0194126.s002.tif]

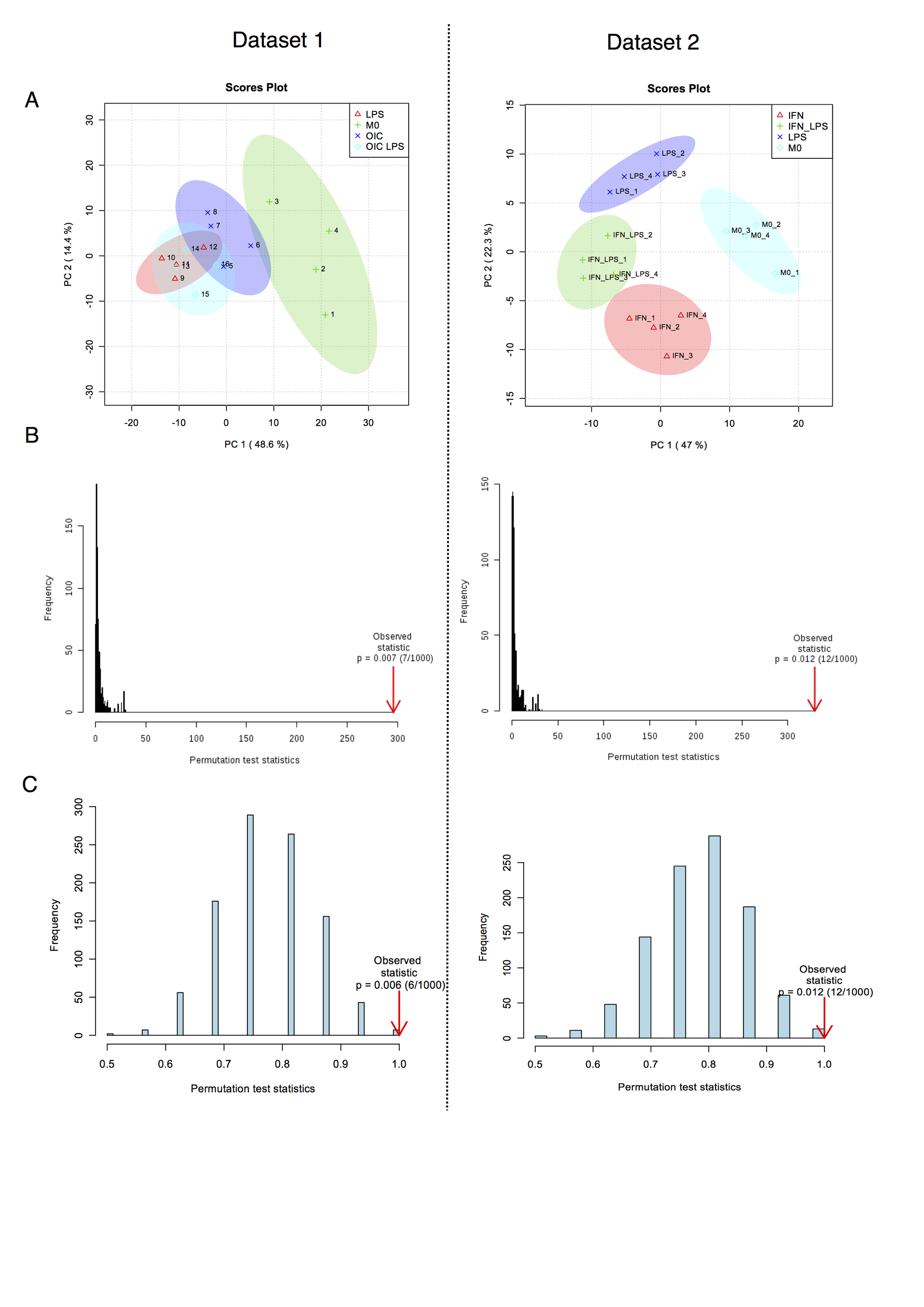

Supplement: S3 Fig — (A) PLS-DA conducted on dataset 1 and 2 (log transformed data). The given conditions are denoted in the inset box. (B) 1000 permutations were run using prediction accuracy during training as well as (C) separation distance (B/W). (TIF) [file pone.0194126.s003.tif]

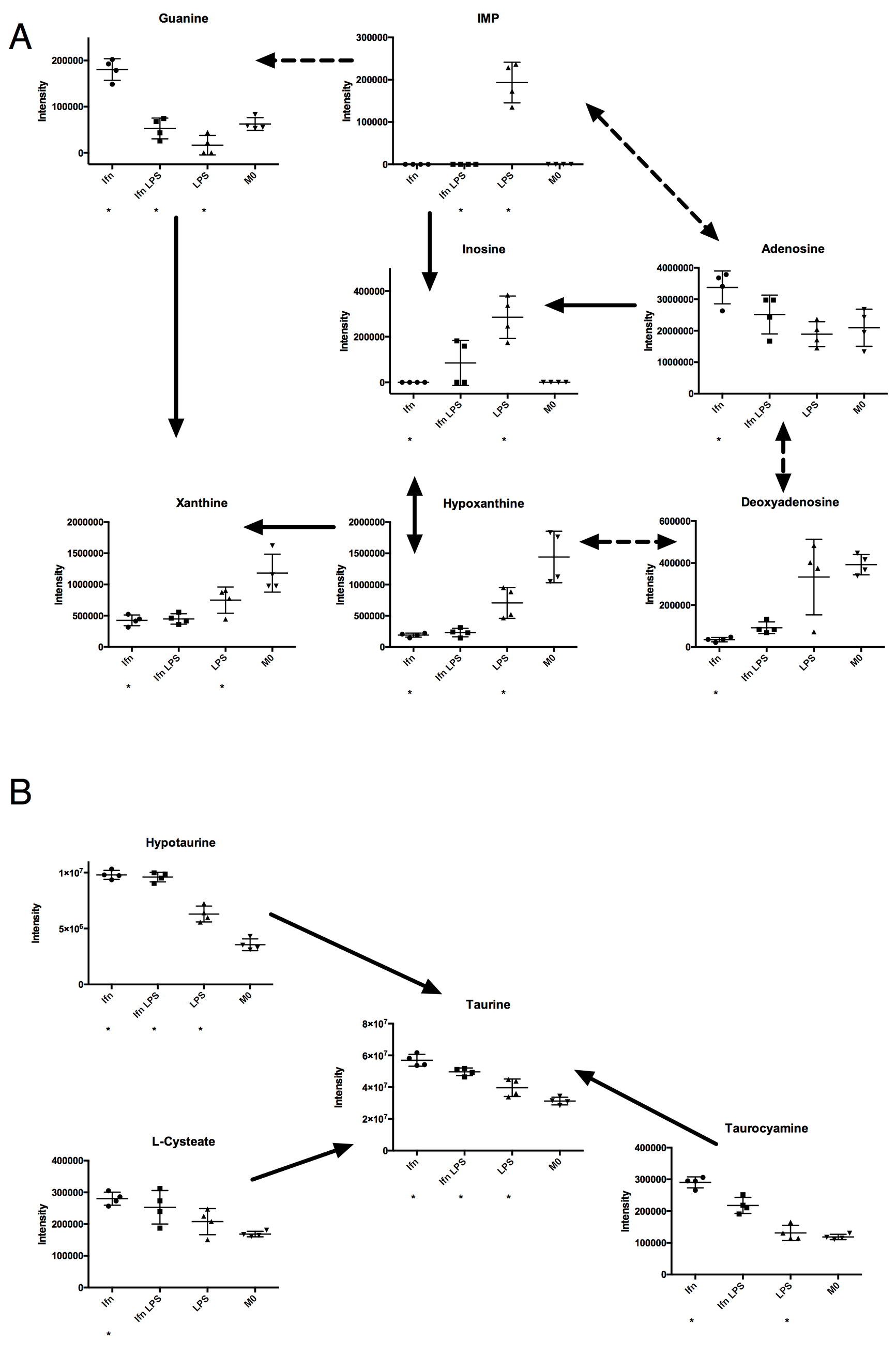

Supplement: S4 Fig — Significance as determined by GLM is denoted by asterisk. Broken lines denote multi-step reactions. (TIF) [file pone.0194126.s004.tif]

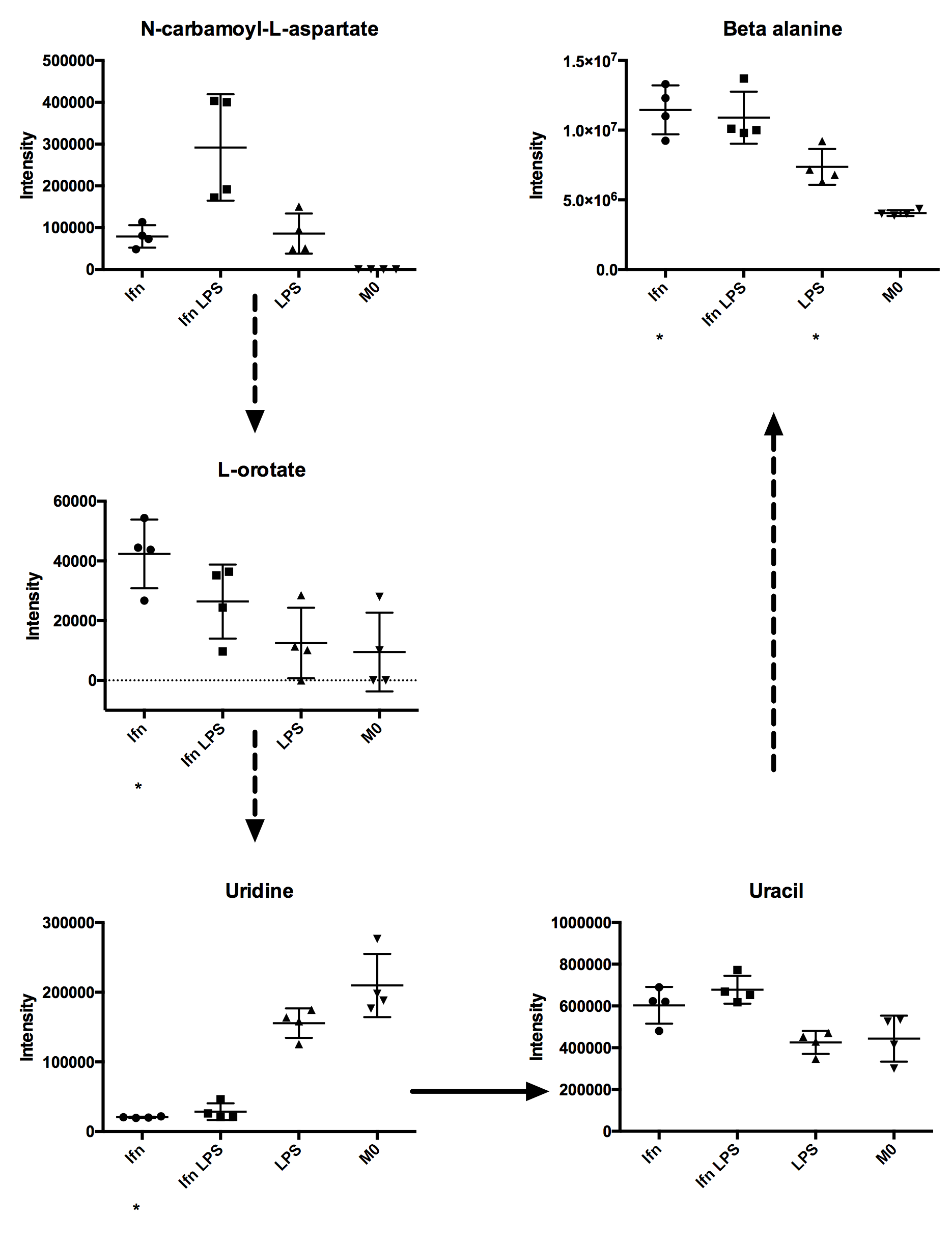

Supplement: S5 Fig — Significance as determined by GLM is denoted by asterisk. Broken lines denote multi-step reactions. (TIF) [file pone.0194126.s005.tif]

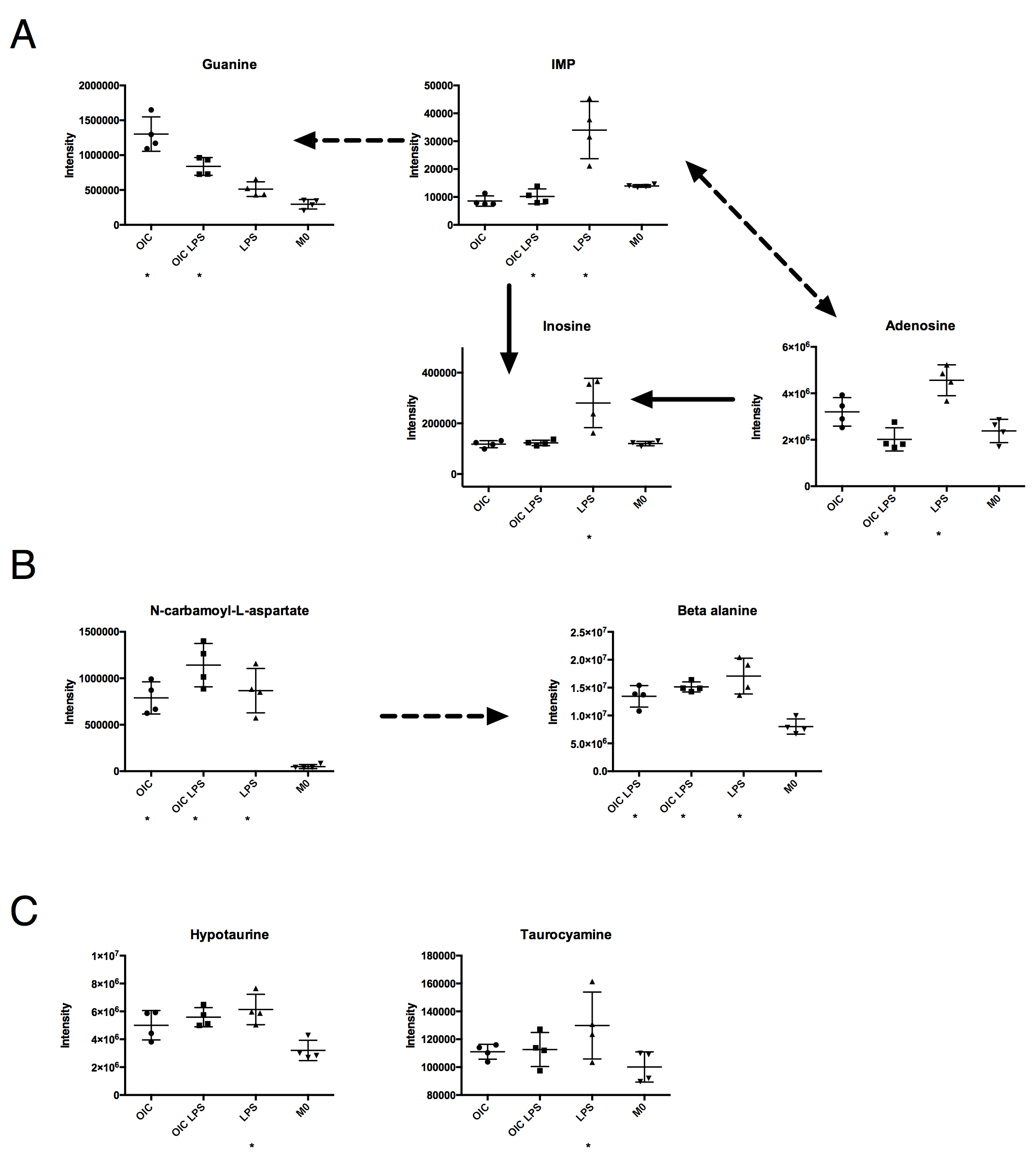

Supplement: S6 Fig — Significance as determined by GLM is denoted by asterisk. Broken lines denote multi-step reactions. (TIF) [file pone.0194126.s006.tif]
